# Oligomeric and Fibrillar α-Synuclein Display Persistent Dynamics and Compressibility under Controlled Confinement

**DOI:** 10.1021/acschemneuro.3c00470

**Published:** 2023-10-20

**Authors:** Katie Lynn Whitcomb, Kurt Warncke

**Affiliations:** Department of Physics, Emory University, Atlanta, Georgia 30322, United States

**Keywords:** synuclein, intrinsically disordered protein, protein dynamics, Parkinson’s disease, electron paramagnetic resonance (EPR)

## Abstract

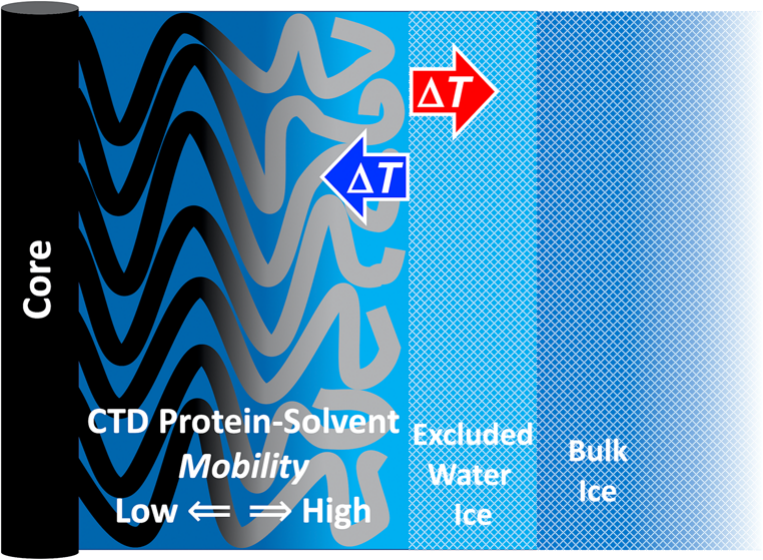

The roles of α-synuclein in neurotransmitter release in brain neurons and in the Parkinson’s disease condition have challenged comprehensive description. To gain insight into molecular mechanistic properties that actuate α-synuclein function and dysfunction, the coupled protein and solvent dynamics of oligomer and fibril forms of human α-synuclein are examined in a low-temperature system that allows control of confinement and localization of a motionally sensitive electron paramagnetic resonance spin probe in the coupled solvent–protein regions. The rotational mobility of the spin probe resolves two distinct α-synuclein-associated solvent components for oligomers and fibrils, as for globular proteins, but with dramatically higher fluidities at each temperature, that are comparable to low-confinement, aqueous-cryosolvent mesophases. In contrast to the temperature-independent volumes of the solvent phases that surround globular and condensate-forming proteins, the higher-fluidity mesophase volume of α-synuclein oligomers and fibrils decreases with decreasing temperature, signaling a compression of this phase. This unique property and thermal hysteresis in the mobilities and component weights, together with previous high-resolution structural characterizations, suggest a model in which the dynamically disordered C-terminal domain of α-synuclein creates a compressible phase that maintains high fluidity under confinement. Robust dynamics and compressibility are fundamental molecular mechanical properties of α-synuclein oligomers and fibrils, which may contribute to dysfunction and inform about function.

## Introduction

The protein, α-synuclein, plays an incompletely specified role in neurotransmitter release in brain neurons.^1^ Dysfunction is associated with Parkinson’s disease (PD) pathology in humans and animal models.^2^ Human α-synuclein is encoded by the SNCA gene and comprises 140 amino acids (14.5 kDa). Monomeric α-synuclein in solution is an intrinsically disordered protein (IDP).^3^ The amphipathic N-terminal domain (NTD; residues 1–60) has α-helix-forming propensity, with 11-mer repeat domains that promote interactions with acidic lipid membranes.^4^ The central residues (61–95) are hydrophobic and constitute the nonamyloid-β component (NAC).^5^ The C-terminal domain (CTD; residues 96–140), which is implicated in protein–protein interactions,^6^ is rich in acidic residues and proline, creating a canonical intrinsically disordered region (IDR). α-Synuclein forms a spectrum of partially structured aggregation states,^7^ including, in increasing degree of order, multimers,^8^ liquid–liquid phase separated (LLPS) condensate,^9,10^ oligomers,^11−13^ and fibrils,^14−18^ all of which feature exposure of the CTD IDR to solvent. Pathological conditions of neurodegeneration are coincident with aggregation of excess α-synuclein, which advances to PD.^2^ Here, we use a low-temperature (*T*) system^19−21^ to introduce fine-tuned, continuously controlled confinement, and localization of an electron paramagnetic resonance (EPR) spin probe in the α-synuclein protein–solvent regions, to reveal fundamental molecular-mechanical and dynamical properties of human α-synuclein oligomers and fibrils. These properties are proposed to contribute to α-synuclein dysfunction and also provide insight into functional features of α-synuclein.

**Figure 1:**
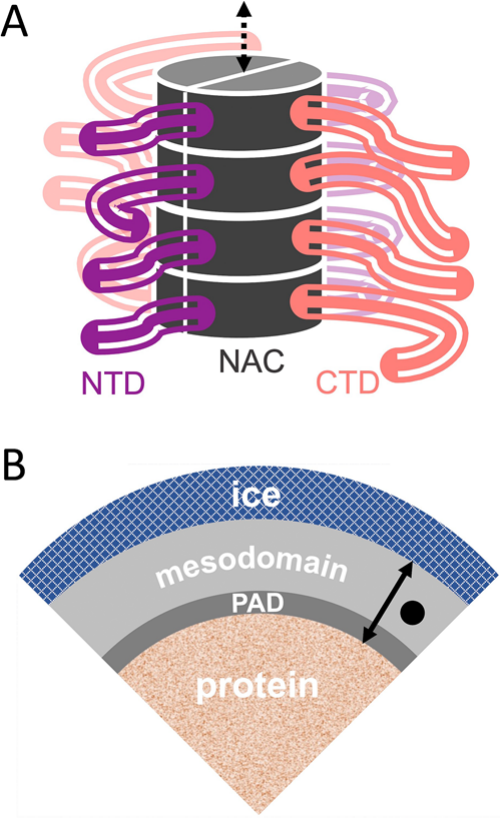
Depictions of the low-temperature frozen solution system and α-synuclein fibril structure. (A) Depiction of four α-synuclein dimer tiers in a fibril based on cryo-EM determinations of ordered, core structures.^17^ NTD (violet) and CTD (rose) protrude from NAC (dark gray) of each monomer on opposed sides of the fibril core. The dashed bidirectional arrow represents continuation of β-strand stacking along the fibril axis. The fibril core diameter, as visualized in transmission electron micrographs, is ∼10 Å. (B) Water-ice delimited solvent structure around folded globular, IDR-containing, and IDP proteins in solutions frozen in the presence of a cryosolvent, such as DMSO.^24^ PAD (mean thickness, 6 Å) and aqueous-DMSO mesodomain (20 Å; corresponding to added 2% v/v DMSO) are shown to scale.^20^ The black circle (diameter, 7 Å) and arrow (length for added 2% v/v DMSO, 26 Å) depict the TEMPOL spin probe and location range, respectively.

The core, β-sheet structure in α-synuclein fibrils has been resolved by solid-state NMR^11,14,15^ and cryo-EM^16,17^ methods. However, the NTD and CTD, which protrude radially along the fibril longitudinal axis, have eluded structural characterization,^12^ owing to their static and dynamic disorder,^11^ respectively (depiction, Figure 1A). α-Synuclein oligomers also display the dynamically disordered CTD on their periphery.^12^ Oligomers range in size from several to approximately 50 monomers with varying NTD/NAC core structures.^11,13^ Small oligomers, formed by lyophilization, have been proposed to have a structure similar to, but less ordered than, fibrils.^11^ Given the crowded intracellular milieu and multiple membrane appression conditions in the neuron presynaptic terminal region, the solvent phase behavior^22^ of the dynamically disordered CTD and its response to confinement^23,24^ in oligomeric and fibrillar forms of α-synuclein are of acute interest.

We have developed a low-*T*, frozen solution system for the study of coupled solvent–protein dynamics (Figure 1B)^19−21^ and their effect on function.^25^ During freezing of aqueous solutions, proteins and solutes are excluded from the advancing ice fronts and come to reside in interstitial regions within the polycrystalline bulk ice.^26^ Freezing also localizes the EPR spin probe, TEMPOL, to the solvent phases directly surrounding the protein, where it reports on the protein-coupled solvent dynamics, through its EPR line shape-detected rotational correlation time (τ_c_), and the relative volumes of the phases, through the normalized component amplitudes (weights, *W*).^19−21^ Globular proteins in frozen solution in the presence of a cryosolvent, added prior to freezing (for example, 0.5–4% v/v dimethyl sulfoxide, DMSO), maintain their structural integrity and display two distinct spin probe mobility components, which represent two fluid phases.^19−21,27^ The slow motional component corresponds to TEMPOL in the protein-associated domain [PAD; akin to the protein hydration layer^28^ of thickness, 6–10 Å], and the fast motional component arises from TEMPOL in an aqueous-cryosolvent phase, or mesodomain, that is located in a layer between the PAD and the polycrystalline ice boundary (Figure 1B). In the complete absence of the added cryosolvent, there is no fluid mesodomain layer (no light gray layer, Figure 1B), and the PAD volume is in immediate contact with the ice boundary, and therefore strongly confined, as shown by restricted TEMPOL tumbling.^19,24^ In contrast to this behavior, we find that oligomeric and fibrillar forms of α-synuclein create a remarkably robust, high-fluidity mesophase around the β-sheet core, even in the absence of a cryosolvent, that persists to low *T* values. This self-fluidizing behavior differs dramatically from soluble globular, IDR-containing, and IDP proteins and provides insights into molecular mechanistic features of α-synuclein that contribute to dysfunction and function.

## Results and Discussion

### Ultrastructure of Oligomeric and Fibrillar α-Synuclein in EPR Samples

**Figure 2:**
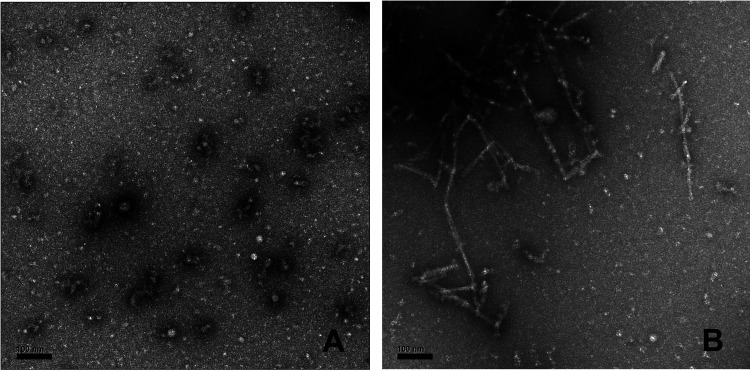
Transmission electron micrographs of α-synuclein from EPR samples: (A) α-synuclein oligomers and (B) α-synuclein fibrils. The prefreeze condition shown corresponds to the EPR sample after mixing of all components and prior to cryotrapping. Samples were prepared for electron microscopy, as described in Materials and Methods. Scale bars, 100 nm.

TEM of the α-synuclein EPR samples, which were prepared from lyophilized powder, showed oligomers with a range of mean diameters from 10 to 20 nm, which aggregate to form larger features up to approximately 50 nm in length (Figure 2A), in common with the previously reported size range (10–90 nm) and symmetries for α-synuclein prepared from lyophilized samples.^11^ Smaller components (10S, 15S) have been isolated by ultracentrifugation and their structures determined by using cryo-EM.^11^ Comparison of the TEM of prefreeze and post-freeze/thaw oligomer samples (Figure S1) shows a close similarity in the sizes of individual and aggregate species. TEM of preformed fibrils of α-synuclein also showed the expected morphology (Figure 2B)^29^ and comparable sample features in the prefreeze and post-freeze/thaw states (Figure S1). The preformed fibrils with lengths of approximately 50–200 nm showed the fundamental width of ∼10 nm, indicating a composition of two β-sheet protofibrils, each of a width of ∼5 nm.^29^ End-to-end, crossed, and aligned fibril species were present, along with a minor population of smaller fragments. Overall, the morphologies of α-synuclein oligomers and fibrils in the EPR samples are consistent with ultrastructures previously characterized by using cryo-EM and solid state NMR.^11,13,29^ The freeze/thaw samples do not significantly differ structurally from the prefreeze states, which supports interpretations of the EPR spin probe results in terms of the visualized and reported oligomer and fibril structures.

### Temperature Dependence of the TEMPOL EPR Spectrum in Frozen Solution Samples of Oligomeric and Fibrillar α-Synuclein

**Figure 3:**
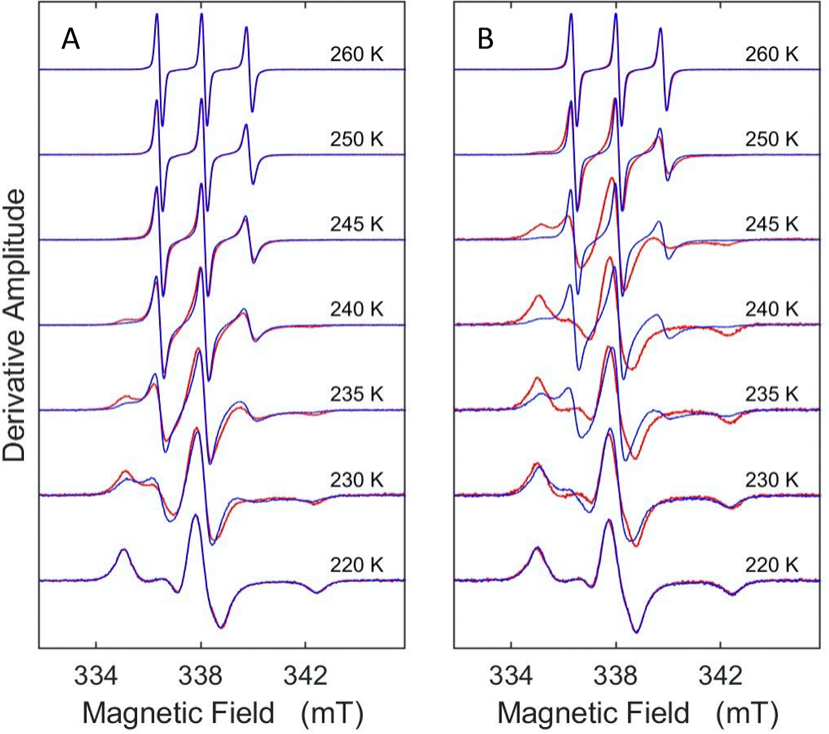
Temperature dependence of the TEMPOL EPR spectrum in the presence of α-synuclein. The spectra show thermal hysteresis for increasing (red spectra) and decreasing (blue spectra) directions of sequential temperature change. (A) α-Synuclein oligomers. (B) α-Synuclein fibrils. The spectra are normalized to the central peak-to-trough amplitude.

EPR spectra of TEMPOL in frozen solution samples of oligomeric and fibrillar α-synuclein in the range of increasing *T* from 220 to 265 K show a common progression from the rigid-limit, broad, powder-pattern line shape to rapid tumbling, motionally narrowed line shape (Figure 3).^21^ The spectra also display a thermal hysteresis, dependent upon the direction of *T* change during the collection of the series of EPR spectra, first increasing from 220 **→** 265 K and then decreasing from 265 **→** 220 K. The spectra within the hysteresis *T* range of 235–250 K indicate lower mobility for the spin probe (broader line shape) when measured for increasing *T*, relative to the spectra at the same *T* value, measured in the direction of decreasing *T*. The extent of the hysteresis is enhanced, and the *T* range is increased, for fibrils, relative to oligomers. At each *T* value, the EPR spectra, and therefore, the hysteresis, are independent of sample holding time. This is shown by the preservation of the EPR line shape and amplitude for 33 min, which is approximately 10-fold longer than typical settling and spectrum acquisition times at each *T* value (Figures S2 and S3, increasing *T*; Figures S4 and S5, decreasing *T*). The hysteresis is also reproducible upon repeated thermal cycling. The sustained low-*T* spin probe mobility and thermal hysteresis have not been observed in previous spin probe studies of soluble globular, IDR-containing, and IDP classes of proteins in the frozen aqueous solution system^19,24^ and are therefore unique to oligomeric and fibrillar α-synuclein.

### Temperature Dependence of the TEMPOL Mobilities and Component Weights

**Figure 4:**
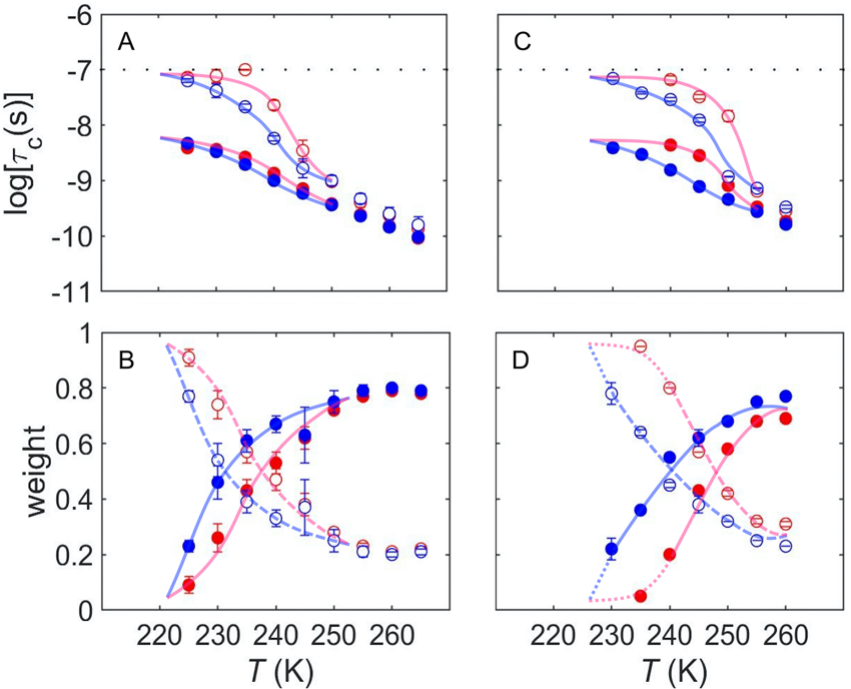
Temperature dependence of the rotational correlation time of TEMPOL and normalized mobility component weights for α-synuclein. (A, B) α-Synuclein oligomers. (C, D) α-Synuclein fibrils. In each panel, solid circles represent the fast component (log τ_c,f_, *W*_f_) and open circles represent the slow component (log τ_c,s_, *W*_s_). Directions of sequential temperature change are indicated by red (increasing) and blue (decreasing). Thermal hysteresis loops are indicated by curves. Lines in panels (C) and (D) are solid (data range for *W*_f_), dashed (data range for *W*_s_; related to *W*_f_ as *W*_s_ = 1 – *W*_f_), and dotted (extrapolations). The horizontal line in panels (A) and (C) represents the upper limit on log τ_c_ for detection of tumbling motion. Error bars represent standard deviations for three (oligomers) and two (fibrils) separate determinations.

Simulations of the α-synuclein EPR line shapes reveal two mobility components: relatively slow (mobility, log τ_c,s_; normalized amplitude or weight, *W*_s_) and fast (log τ_c,f_, *W*_f_) (experimental and simulated spectra overlays, Figures S3–S6; corresponding simulation parameters, Tables S1–S4). The general patterns of the *T*-dependences of the log τ_c_ and *W* values of the two components are comparable for oligomer and fibril samples (Figure 4). In the high *T* range of ≥255 K, the weight of the fast component is dominant, but as *T* is lowered, the slow component grows in proportion and becomes dominant below 235 K. Hysteresis in the *T*-dependence of log τ_c_ and *W* plots is demarcated by loops (inscribed, Figure 4). Both log τ_c_ and *W* parameters exhibit greater hysteresis for fibrils relative to oligomers. At each *T* value, the direction of ascending *T* is characterized by lower mobility for each component (higher log τ_c_ value) and a lower *W*_f_ value. For both oligomers and fibrils, the temperature ranges of the hysteresis loops for log τ_c,s_, log τ_c,f_, and *W* values are coincident, indicating a common origin.

### Enhanced Solvent Phase Dynamics around α-Synuclein Oligomers and Fibrils

**Figure 5:**
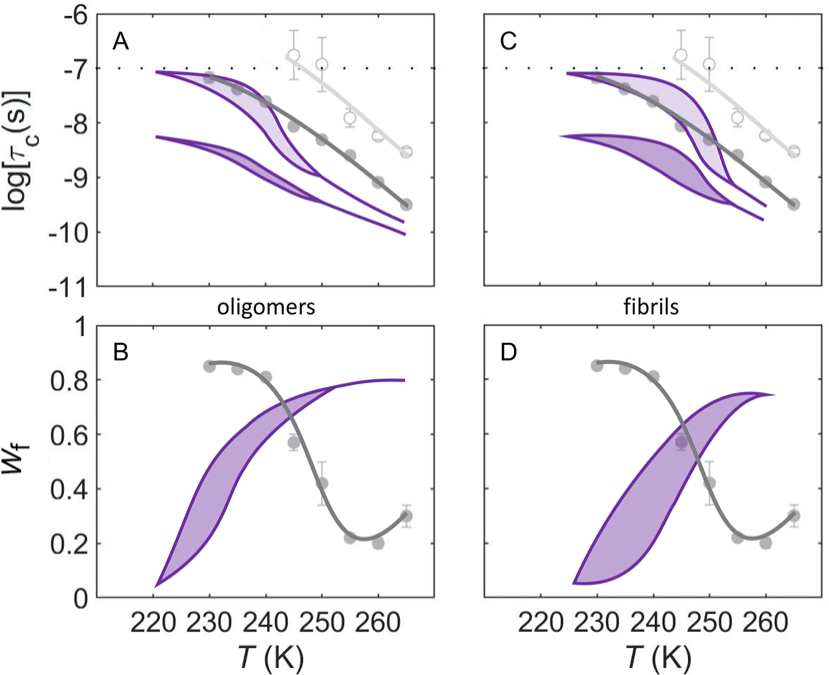
Temperature dependence of the rotational correlation times of TEMPOL and normalized mobility component weights for α-synuclein and the soluble globular protein, EAL. In all panels, α-synuclein (violet) and EAL (gray) data^19,20^ correspond to the absence of cryosolvent. (A, B) α-Synuclein oligomers and EAL. (C, D) α-Synuclein fibrils and EAL. EAL data are shown for the slow component (log τ_c,s_, *W*_s_; light gray open circles, curves) and fast component (log τ_c,f_, *W*_f_; dark gray filled circles, curves). Hysteresis loops for α-synuclein are obtained from Figure 4 and are represented as filled violet (light shade, slow component; dark shade, fast component). Redundant data for *W*_s_ are not shown in panels (B) and (D) (*W*_s_ = 1 – *W*_f_). The horizontal line in panels (A) and (C) represents the upper limit on log τ_c_ for detection of tumbling motion. Error bars for EAL data represent standard deviations for three separate determinations.

Soluble globular proteins display common *T*-dependent solvent-coupled protein dynamics in the low-*T* system, which lead to the model presented in Figure 1B.^24^ To assess the protein-coupled solvent dynamics around α-synuclein oligomers and fibrils, we compared their *T*-dependences with the *T*-dependence of the representative soluble globular protein, ethanolamine ammonia-lyase (EAL). Remarkably, the spin probe mobilities of both slow and fast components are dramatically enhanced for α-synuclein oligomers and fibrils, in comparison with the corresponding values for EAL (lower, more negative values of log τ_c,s_ and log τ_c,f_; Figure 5A,C),^19,24^ for both arms of the α-synuclein hysteresis loops. Another significant difference between α-synuclein and EAL is the inversion of the *W* versus *T* dependence (Figure 5B,D; only *W*_f_ is plotted, for clarity, owing to the relation *W*_s_ = 1 – *W*_f_; *W*_s_ would be symmetrically inverted about the value, 0.5). For both oligomers and fibrils, the fast, *W*_f_ component dominates at high *T* values (≥250 K) and becomes the minor component below ∼235 K, whereas the opposite holds for globular proteins.^24^ The *W*_s_ and *W*_f_ values represent the relative volumes of the PAD and mesodomain phases.^19,20^ Therefore, unlike globular proteins, α-synuclein oligomers and fibrils create a high-fluidity phase that diminishes in volume with decreasing *T* value.

### Cryosolvent-like Solvent Phase Dynamics Are Created by α-Synuclein Oligomers and Fibrils

**Figure 6:**
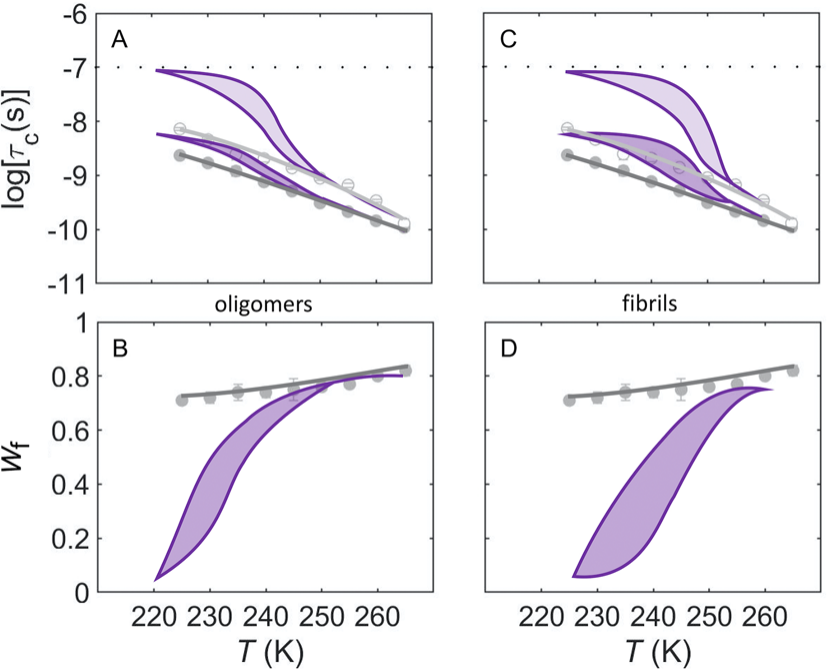
Temperature dependence of the rotational correlation times of TEMPOL and normalized mobility component weights for α-synuclein, and the soluble globular protein EAL, in the presence of cryosolvent. The curves for α-synuclein (violet) are the same as presented in Figure 4. EAL (gray) data^19,20^ correspond to the condition of added 2% v/v DMSO cryosolvent. (A, B) α-Synuclein oligomers and EAL. (C, D) α-Synuclein fibrils and EAL. EAL data are shown for the slow component (log τ_c,s_, *W*_s_; light gray open circles, curves) and fast component (log τ_c,f_, *W*_f_ ; dark gray filled circles, curves). Hysteresis loops for α-synuclein are obtained from Figure 4 and represented as filled violet (light shade, slow component; dark shade, fast component). Redundant data for *W*_s_ are not shown in panels (B) and (D) (*W*_s_ = 1 – *W*_f_). The horizontal line in panels (A) and (C) represents the upper limit on log τ_c_ for detection of tumbling motion. Error bars for the EAL data represent standard deviations for three separate determinations.

To determine the relative degree of fluidity enhancement observed for α-synuclein oligomers and fibrils under the high-confinement conditions in the absence of a cryosolvent (Figure 4), we compared the α-synuclein behavior to EAL samples, in which 2% DMSO was added, to create the aqueous-DMSO mesodomain around EAL and its hydration layer (PAD) (Figure 1B). Strikingly, for *T* values down to ∼245 K, the mobility of the α-synuclein fast component for oligomers and fibrils is comparable to the fast, aqueous-DMSO mesodomain mobility component for EAL, which maintains fluidity at cryogenic temperatures (Figure 6A,C).^20,24^ Significantly, for EAL and other globular proteins surrounded by the layer of aqueous-DMSO cryosolvent mesodomain, the *W*_f_ and *W*_s_ values are essentially constant over the *T* range of 225–265 K (Figure 6B,D),^19,20,24^ which indicates that the PAD and mesodomain volumes are stable, consistent with the relative incompressibility of these fluid phases. In contrast, the *W*_f_ and *W*_s_ values in α-synuclein oligomer and fibril samples vary strongly with *T* (Figure 6B,D). Overall, the results suggest that the highly dynamic phase created by α-synuclein oligomers and fibrils emulates the fluidity of an aqueous-cryosolvent phase, but not its incompressibility.

### Model for Confinement-Resistant Dynamics of α-Synuclein Oligomers and Fibrils

We considered the origins of the unexpected phase dynamics of α-synuclein oligomers and fibrils, starting from the structural and dynamical insights provided by cryo-EM and NMR techniques.^11,14−17^ In α-synuclein fibrils, the structurally well-defined positions of the central β-strand pair of interacting α-synuclein monomers at each layer along the fibril axis are oriented so that the structurally undefined, disordered IDRs of the NTD (residues ∼1–40) and CTD (residues ∼96–140) protrude radially (depiction, Figure 1A).^14,15,17^ Disorder is static in the NTD and dynamic in the CTD.^14,30^ Oligomers of α-synuclein vary in size and include species with annular (tubular) and amorphous (globular) morphologies but have a common NTD/NAC core structure, which is surrounded by the dynamically disordered CTD.^12^ We thus propose a model, in which the aqueous cryosolvent-like properties of oligomeric and fibrillar α-synuclein arise principally from the dynamically disordered CTD IDR. Disorder in the CTD originates from the defeat of intra- and interpeptide interactions by the combination of high negative charge density from Asp and Glu residues and chain-kinking by Pro residues,^12^ which collaterally disrupt the protein and associated aqueous solvent structure.

**Figure 7:**
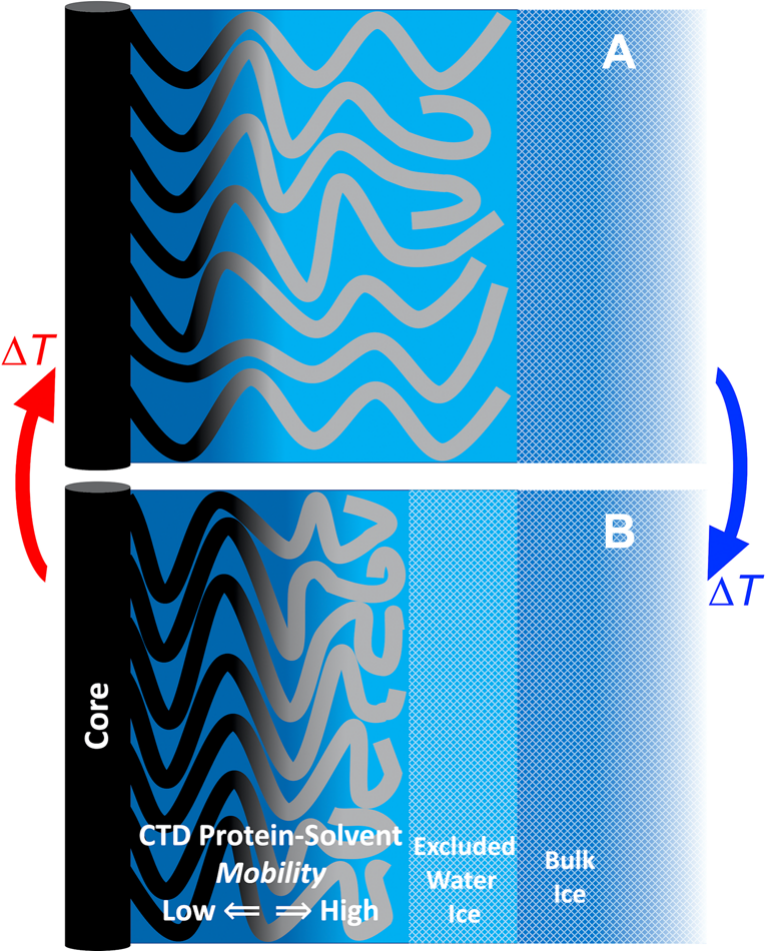
Model for the confinement-resistant dynamics and thermal hysteresis in oligomeric and fibrillar forms of α-synuclein in a frozen aqueous solution system. The depiction projects the CTD and underlying NTD/NAC domains of the fibril (Figure 1A) or oligomer arrays onto two-dimensions. The CTD polypeptide chains extend along a coordinate (horizontal) from the anchoring NTD/NAC region (β-sheet core, black). The dynamics of the CTD and neighbor regions are represented by black (low mobility, near anchor point, left), transitioning to gray (higher mobility, right). The corresponding dynamics of the surrounding aqueous solvent are represented as dark blue (slow) and light blue (fast). The change from panel (A) to (B) represents the compaction of the CTD region in response to a temperature decrease (blue arrow) for *T* ≤ 250 K, during which water from the CTD region is extruded and adds to an external ice crystalline layer (light blue, hatched) adjacent to the bulk ice (darker blue, hatched). In the reverse direction of the temperature increase (red arrow), the extruded water-ice melts and infuses the CTD region.

Our model for the *T*-dependent dynamical properties of α-synuclein oligomers and fibrils is presented in Figure 7. The dominance of *W*_f_ at high *T* values is explained by the location of TEMPOL in a large-volume fluid mesophase created by the solvent-coupled, dynamically disordered region of the CTD IDR. The lower mobility fraction, represented by *W*_s_, arises from TEMPOL in a PAD created by relatively low-mobility regions of the CTD and the neighboring solvent-accessible protein surface of the NAC and NTD regions (Figure 1A). As *T* is decreased, water exits the solvent-CTD phase and crystallizes at the ice boundary on the periphery of the CTD IDR region, leading to progressive, increased confinement and compression of the disordered CTD region (Figure 7). The compression of the CTD causes a decrease in the volume of the solvent region with the higher TEMPOL mobility and, therefore, redistribution of TEMPOL and a decrease in *W*_f_. This leads to a compensatory increase in the relative proportion of *W*_s_, which corresponds to the relocation of TEMPOL to the PAD regions. This leads to the dominance of *W*_s_ at low *T* values. The relatively small proportion of TEMPOL-detectable PAD at higher *T* values in the α-synuclein samples is consistent with the absence of a PAD in unfolded, disordered polypeptide chains under low confinement^24^ and the absence of the low-mobility, protein-bound class of water in disordered proteins, including α-syn, observed by using Overhauser dynamic nuclear polarization.^31^

The model in Figure 7 also provides a rationale for the thermal hysteresis in log τ_c_ and *W* values. During the *T* decrease, the mobile, disordered CTD harbors water and destabilizes additional ice formation at the CTD-ice boundary. Water expelled from the collapsing IDR crystallizes along the encroaching ice front. Upon stepwise return of the sample to higher *T* values, this ice layer resists melting and repopulation of the compressed CTD solvent region volume. At each *T*, a higher *T* value is required to melt the excluded boundary ice to restore the same CTD volume and, therefore, *W*_f_ value. We explain differences in the degree and absolute *T*-range of oligomer and fibril hysteresis in terms of their structures. The anchoring of the CTD to the β-sheet core offsets the protruding IDRs. The regularity of the opposed (rotation by 180° about the fibril axis), radial protrusion of CTD pairs in the fibril array, and consequent larger accommodating volume for the polypeptide chains upon compression, relative to a more planar, 2-dimensional CTD packing in oligomers, amplifies the degree of hysteresis for fibrils relative to oligomers (Figure 4). The larger accommodating volume for the fibrillar CTD compression also rationalizes the increase in the mean *T* position of the hysteresis loop by ∼7 K, relative to oligomers (Figure 4). The results further suggest that the anchored arrangement prevents global reorientational freedom of the CTD, thwarting the formation of a condensate phase, or LLPS, characteristic of free IDPs, such as β-casein and protamine, in the low-*T* system.^24^

## Conclusions

The results show that robust, confinement-resistant dynamics and compressibility are fundamental molecular-mechanical properties manifested by the oligomeric and fibrillar states of α-synuclein. These properties, proposed to originate predominantly from the CTD regions and their interaction with solvent, are distinct from those of globular proteins, condensate-forming IDPs, and proteins with IDR domains.^24^ Restriction of the α-synuclein monomer units by the ordered fibril structure, and to a lesser extent, by the oligomer structures, so that the disordered domains are displayed without veiling by non-neighbor self-interactions, is a key feature of generating the unique dynamics. The special dynamical properties of the CTD region may contribute to α-synuclein dysfunction and function. α-Synuclein dysfunction is associated with disruption and permeabilization of neuronal cellular plasma and mitochondrial membranes^12,32^ by toxic oligomer species.^11,33^ Immobilization of the CTD by the polyphenolic epigallocatechin gallate (EGCG) eliminates the membrane permeabilization and cytotoxicity of α-synuclein.^34^ Computational modeling shows localized disruption of lipid bilayer integrity by the CTD during the process of NTD/NAC-guided insertion of α-synuclein into the membrane.^35^ Thus, the confinement-resistant dynamics of the CTD, displayed on the oligomer or fibril periphery, are proposed to promote membrane destabilization under appressed, direct-contact conditions. The native function of α-synuclein is associated with the confined neuron axon terminal region and the synaptic vesicle liquid phase.^36^ The structure of native, membrane-associated α-synuclein in the functional monomer, or small copy-number multimer states, is α-helical,^37^ with a structurally uncharacterized, disordered CTD that extends from the attachment point at the lipid bilayer. In the neuron bouton, presynaptic vesicles are decorated with α-synuclein and tightly packed in the synaptic vesicle cluster.^9,36^ Under this high-confinement condition, we propose that the synaptic vesicle liquid phase^36^ is maintained by the dynamic CTD. In agreement, synaptic vesicles in α-synuclein knockout mice are observed to have a higher packing density.^38^ In the next stage of the in vivo process, the release of neurotransmitter into the synaptic cleft and fusing of the appressed synaptic vesicle and presynaptic membranes under high confinement would also be facilitated by the persistent mobility of the α-synuclein CTD. Dynamic disorder in terminal peptide segments extending from an ordered β-sheet core is also found in fibrils of the huntingtin protein (Huntington’s disease)^39^ and prion protein (Creutzfeld–Jakob disease).^40^ Confinement-resistant dynamic disorder of anchored IDR components of proteins may thus be a general principle that contributes to dysfunction, in addition to revealing traits relevant to native function. Toward this, the dynamical behavior of other fibril-forming IDR proteins and monomeric α-synuclein are currently being investigated in the low-*T* mesodomain system.

## Methods and Materials

### EPR Sample Preparation

All chemicals were obtained from commercial sources. Oligomeric human α-synuclein was obtained from the lyophilized powder (rPeptide, Athens, Georgia, US; P/N S-1001-2) and was initially suspended at 1 mg/mL in water (deionized; resistivity, 18.2 MΩ cm), with brief vortex mixing (10–15 s). Preformed fibrils (rPeptide, Athens, Georgia, US; P/N ASF-1001-1) of human α-synuclein were obtained as a liquid (10 mg/mL). EPR samples contained 0.5 mg/mL α-synuclein in 10 mM potassium phosphate buffer (pH 7.4), with TEMPOL added from freshly prepared stock solution to 0.02 mM, for a total volume of 0.3 mL. Samples were transferred to EPR tubes (4 mm outer diameter; Wilmad-LabGlass, Buena, NJ, US), frozen by immersion in isopentane at 140 K, and stored in liquid nitrogen prior to measurements.^21^

### Continuous-Wave EPR Spectroscopy

X-band CW-EPR measurements were performed by using a Bruker E500 ElexSys EPR spectrometer and ER 4123SHQE X-band cavity resonator with temperature calibration and control, as described,^41^ by using the following acquisition parameters: microwave frequency, 9.5 GHz; microwave power, 0.2 mW; magnetic field modulation, 0.2 mT; modulation frequency, 100 kHz. Four to eight spectra were averaged at each temperature.

### Transmission Electron Microscopy

TEM samples corresponding to the prefreeze condition were prepared from the EPR sample volume after mixing of all components and prior to cryotrapping. TEM samples corresponding to the post-freeze/thaw condition were prepared from a volume removed from the EPR sample tube, following EPR measurement and rethawing. EM grids (thick carbon, 400 mesh, copper grids; Electron Microscopy Sciences, Hatfield, PA, US) were cleansed by glow discharge. For each sample, a grid was placed on top of a 60 μL droplet for 5 min and then transferred to the top of a droplet of 2% uranyl acetate for 1 min. Grids were dried thoroughly before imaging. Images were obtained by using a JEOL JEM-1400 instrument (Figure 2) or Hitachi HT-7700 TEM (Figure S1) operating at 80 kV.

### EPR Line Shape and EPR Simulations

The EPR spectrum from the randomly oriented TEMPOL spin probe arises from the interactions of the unpaired electron spin (*S* = 1/2) with the external magnetic field (Zeeman interaction; defined by the **g** tensor) and the nitroxide ^14^N nuclear spin (*I* = 1) through the hyperfine interaction (defined by the ^14^N hyperfine tensor). At X-band microwave frequencies and magnetic fields, the ^14^N hyperfine interaction is dominant, and the three spectral features correspond to electron spin–spin transitions (Δ*m*_s_ = ±1/2) among the three ^14^N nuclear spin states, *m*_I_ = 0, ±1.^42^ At higher temperatures, the averaging of the dipolar hyperfine interaction by relatively rapid rotational motion of the spin probe resolves three spectral features, which are separated by the isotropic ^14^N hyperfine coupling constant. Slower rotation at lower temperatures leads to the manifestation of the orientation-dependent dipolar hyperfine interaction, which broadens each *m*_I_ feature. These effects of motion on the TEMPOL line shape^43,44^ are the basis for extraction of the rotational correlation time, τ_c_, and component weights, *W*, by using EPR simulations. Methods for simulating the nitroxide EPR spectra in the low-*T* frozen solution system, by using the program EasySpin^45^ and a common set of **g** and ^14^N hyperfine tensor principle values, have been described in detail.^21^ The correlation times obtained from the simulations are in excellent agreement with those calculated for known solvent viscosity by using the Debye–Stokes–Einstein expression^24^ and correspond to slow (τ_c_ → 10^–7^ s), intermediate, and rapid (τ_c_ → 10^–11^ s) TEMPOL tumbling regimes, which define the X-band motion-detection bandwidth.^43^

## Supporting Information Available

Mean log τ_c_ and *W* values at different *T* values for α-synuclein oligomers for increasing and decreasing *T* change (Tables S1 and S2); mean log τ_c_ and *W* values at different *T* values for α-synuclein fibrils for increasing and decreasing *T* change (Tables S3 and S4); transmission electron micrographs of α-synuclein from oligomer and fibril EPR samples for the prefreezing and post-freeze/thaw conditions (Figure S1); time dependence of the EPR spectrum of TEMPOL in the presence of α-synuclein oligomers for the *T* step increase (Figure S2) and decrease (Figure S4); comparison of initial and final EPR spectra for the time dependence of the EPR spectrum of TEMPOL in the presence of α-synuclein oligomers for the *T* step increase (Figure S3) and decrease (Figure S5); temperature dependence of the TEMPOL EPR spectrum and overlaid two-component EPR simulations for α-synuclein oligomers (Figures S6 and S7); temperature dependence of the TEMPOL EPR spectrum and overlaid two-component EPR simulations for α-synuclein fibrils (Figures S8 and S9) (PDF)
