# Supplementary material for: Oligomeric and Fibrillar α-Synuclein Display Persistent Dynamics and Compressibility under Controlled Confinement
Source: ACS Chem Neurosci. 2023 Oct 20;14(21):3905–12. doi: 10.1021/acschemneuro.3c00470 (PMC10623556; doi:10.1021/acschemneuro.3c00470)
Supplement: Supplementary file 1 — cn3c00470_si_001.pdf [file cn3c00470_si_001.pdf]

Oligomeric and Fibrillar Alpha-Synuclein Display Persistent Dynamics and Compressibility Under Controlled Confinement

Katie Lynn Whitcomb and Kurt Warncke

Department of Physics, Emory University, Atlanta, Georgia 30322

Supporting Information

Table of Contents

SI Figures

Figure S1. Transmission electron micrographs of α-synuclein from oligomer and fibril EPR samples for the pre-freezing and post-freeze/thaw conditions.

Figure S2. Time dependence of the EPR spectrum of TEMPOL in the presence of α-synuclein oligomers, at the representative *T* value of 235 K, after temperature increase.

Figure S3. Comparison initial and final EPR spectra for the time dependence of the EPR spectrum of TEMPOL in the presence of α-synuclein oligomers, at the representative *T* value of 235 K, after temperature increase.

Figure S4. Time dependence of the EPR spectrum of TEMPOL in the presence of α-synuclein oligomers, at the representative *T* value of 235 K, after temperature decrease.

Figure S5. Comparison initial and final EPR spectra for the time dependence of the EPR spectrum of TEMPOL in the presence of α-synuclein oligomers, at the representative *T* value of 235 K, after temperature decrease.

Figure S6. Temperature dependence of the TEMPOL EPR spectrum in the presence of α-synuclein and overlaid two-component EPR simulations: α-synuclein oligomers, for increasing sequential temperature change.

Figure S7. Temperature dependence of the TEMPOL EPR spectrum in the presence of α-synuclein and overlaid two-component EPR simulations: α-synuclein oligomers, decreasing sequential temperature change.

Figure S8. Temperature dependence of the TEMPOL EPR spectrum in the presence of α-synuclein and overlaid two-component EPR simulations: α-synuclein fibrils, for increasing sequential temperature change.

Figure S9. Temperature dependence of the TEMPOL EPR spectrum in the presence of α-synuclein and overlaid two-component EPR simulations: α-synuclein fibrils, for decreasing sequential temperature change.

SI Tables

Table S1. Mean logτ_c and W values at different T values for α-synuclein oligomers, for data collection in the direction of increasing T.

Table S2. Mean logτ_c and W values at different T values for α-synuclein oligomers, for data collection in the direction of decreasing T.

Table S3. Mean logτ_c and W values at different T values for α-synuclein fibrils, for data collection in the direction of increasing T.

Table S4. Mean logτ_c and W values at different T values for α-synuclein fibrils, for data collection in the direction of decreasing T.

SI Figures

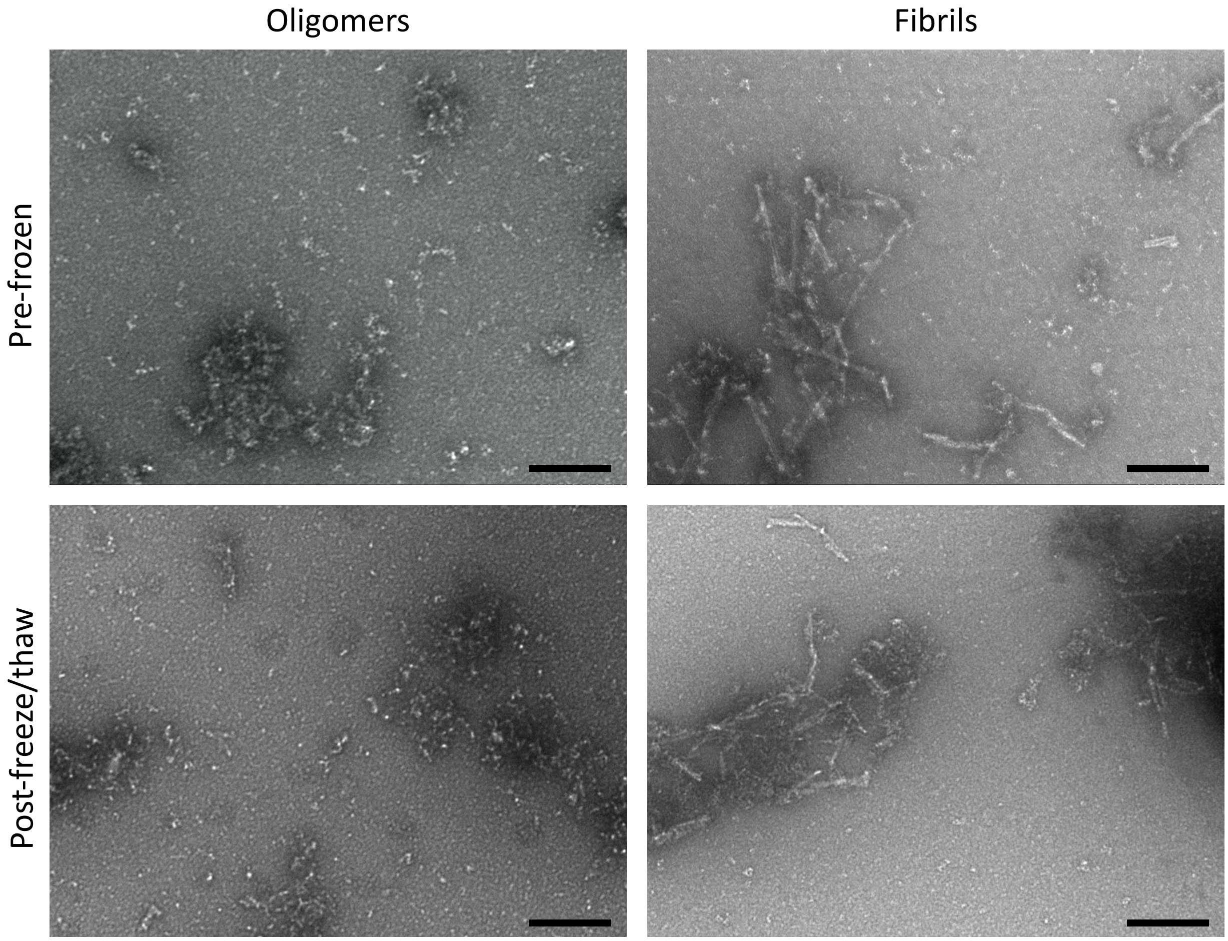

Figure S1. Transmission electron micrographs of α-synuclein from oligomer and fibril EPR samples for the pre-frozen and post-freeze/thaw sample conditions. The pre-frozen TEM sample condition corresponds to the EPR sample after mixing of all components and prior to cryotrapping. The post-freeze/thaw TEM sample condition corresponds to a volume removed from the EPR sample tube, following cryotrapping, EPR measurement, and rethawing. Details of the procedures are described in Materials and Methods. Scale bar, 200 nm.

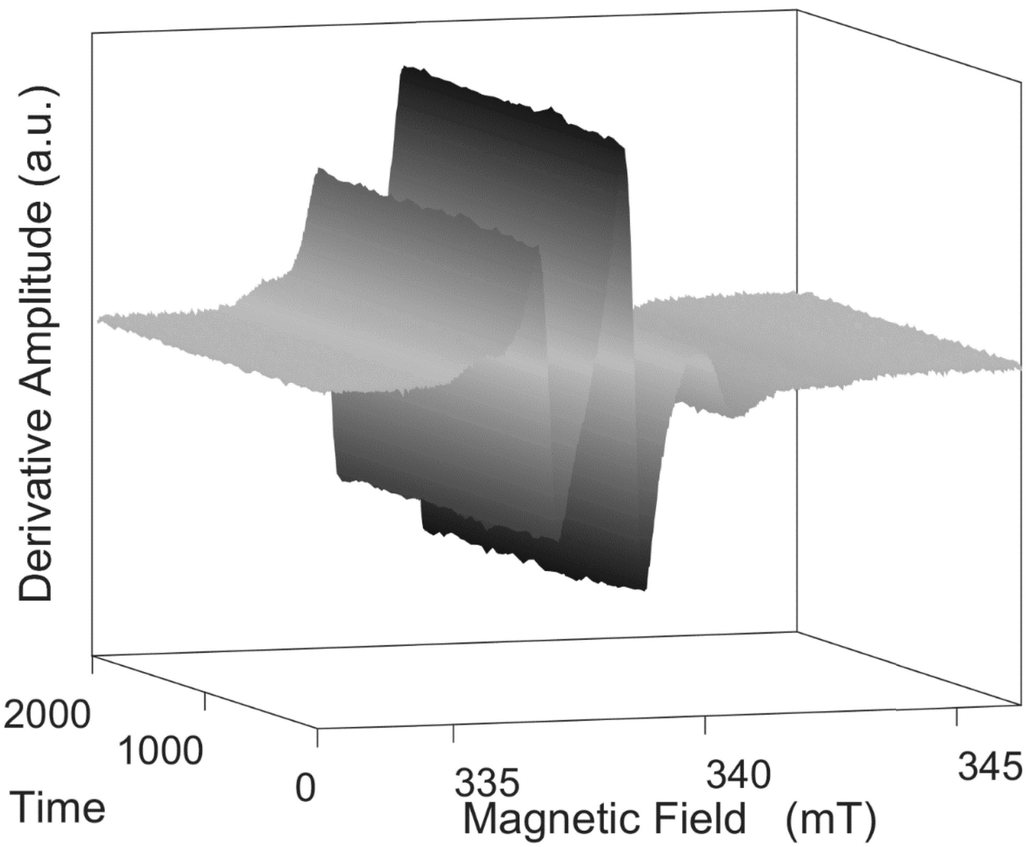

Figure S2. Time dependence of the EPR spectrum of TEMPOL in the presence of α-synuclein oligomers, at the representative *T* value of 235 K, after temperature increase. EPR conditions and data acquisition procedures are as described in Materials and Methods. The time sequence over 2×10^3 s (33 min) of TEMPOL EPR spectra was collected following *T*-step from 225 K to 235 K.

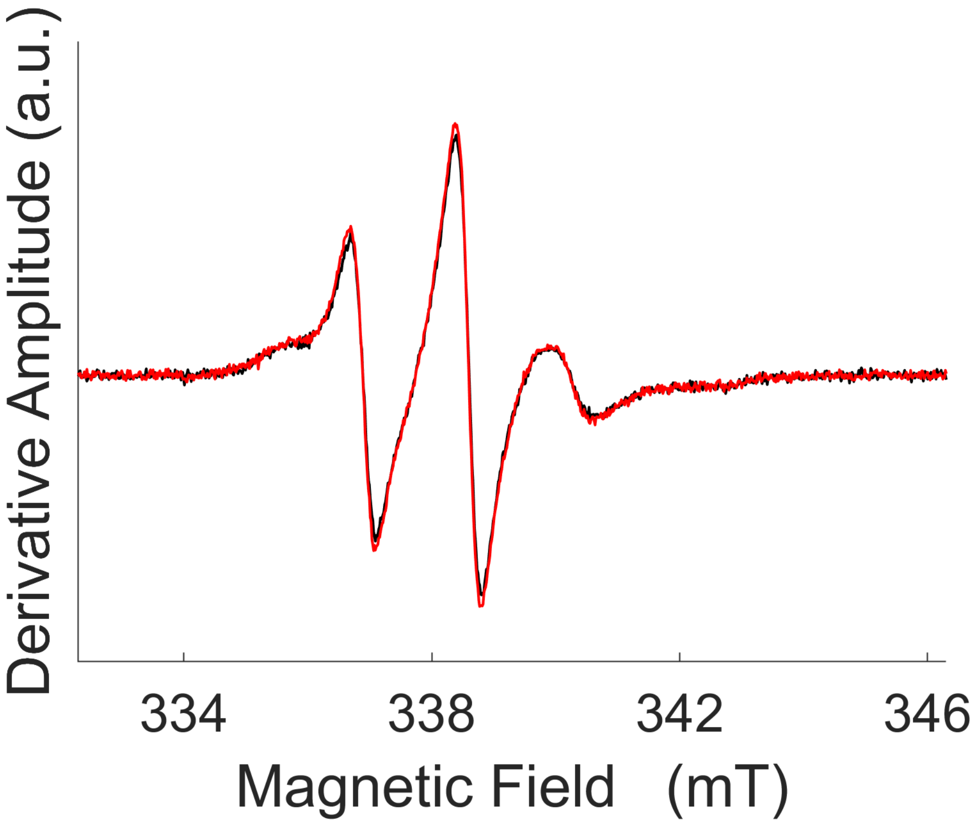

Figure S3. Comparison initial and final EPR spectra for the time dependence of the EPR spectrum of TEMPOL in the presence of α-synuclein oligomers, at the representative *T* value of 235 K, after temperature increase. Overlaid TEMPOL EPR spectra, corresponding to average of initial four EPR spectra (black) and average of final four EPR spectra (red) from the time dependent series of spectra in Fig. S1. EPR conditions and data acquisition procedures are as described in Materials and Methods.

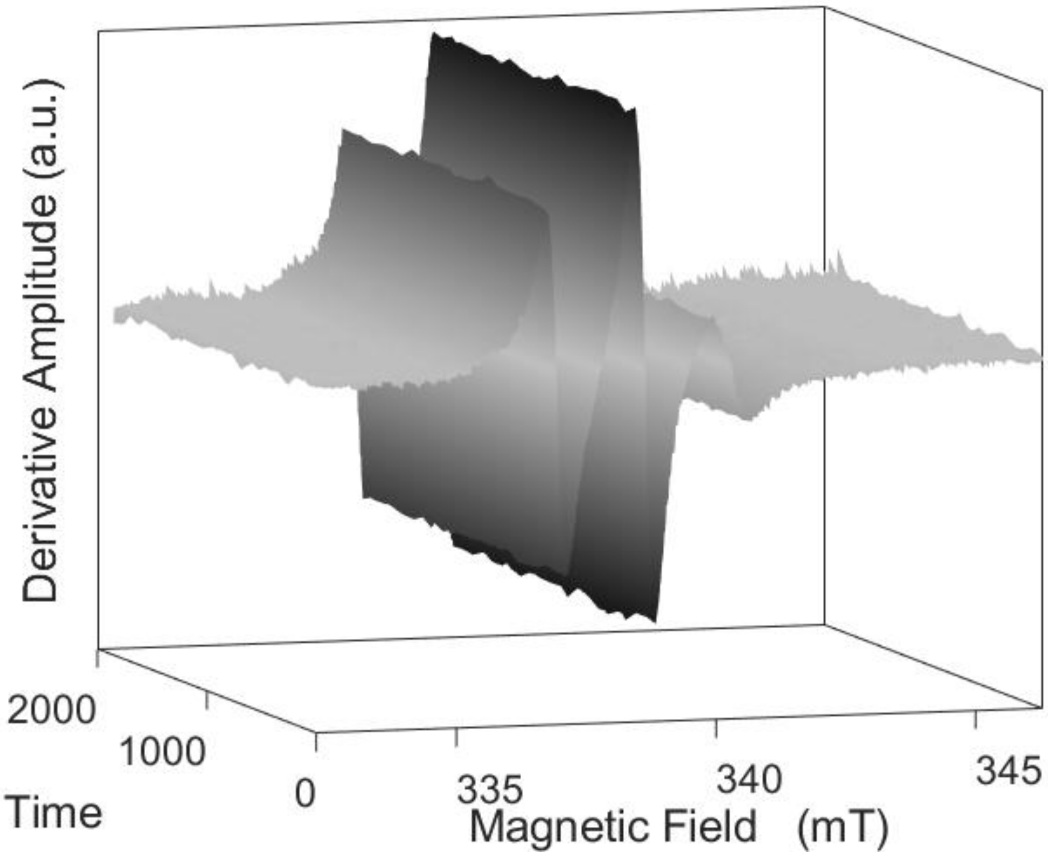

Figure S4. Time dependence of the EPR spectrum of TEMPOL in the presence of α-synuclein oligomers, at the representative *T* value of 235 K, after temperature decrease. EPR conditions and data acquisition procedures are as described in Materials and Methods. The time sequence over $2{\times}10^3$ s (33 min) of TEMPOL EPR spectra were collected following *T*-step from 245 K to 235 K.

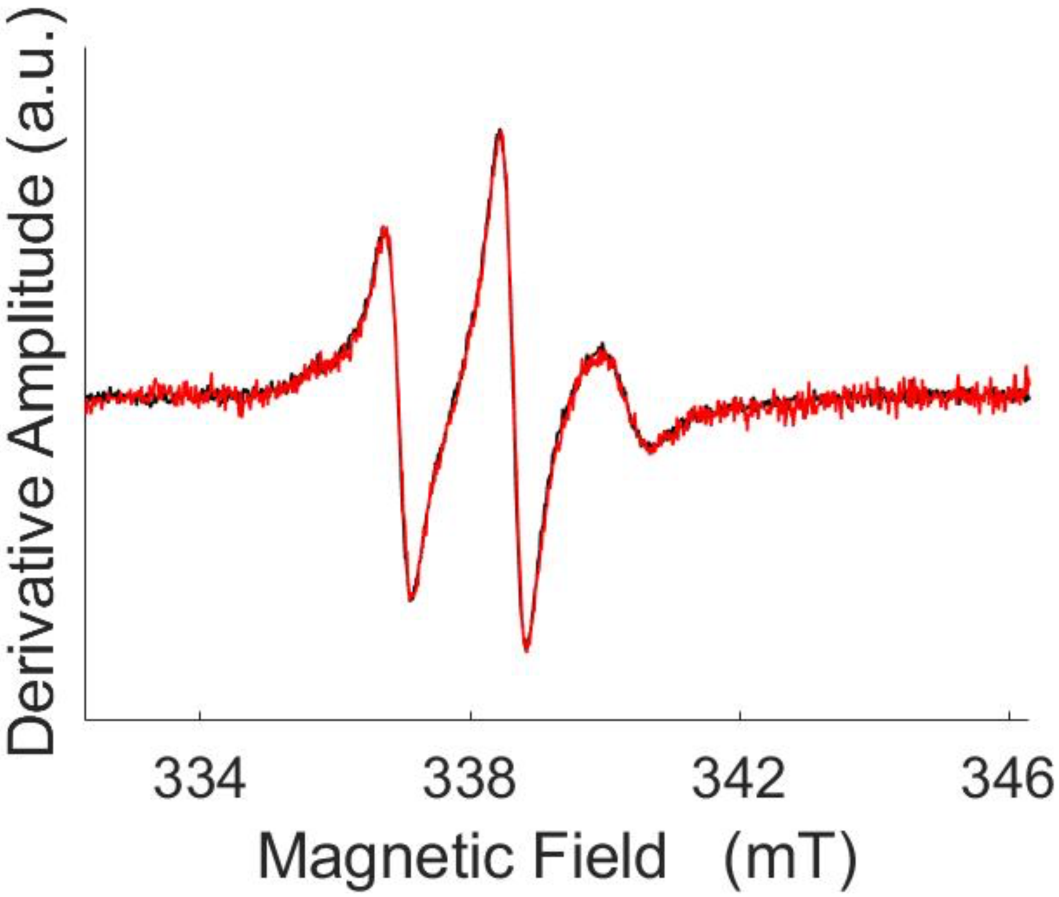

Figure S5. Comparison initial and final EPR spectra for the time dependence of the EPR spectrum of TEMPOL in the presence of α-synuclein oligomers, at the representative *T* value of 235 K, after temperature decrease. Overlaid TEMPOL EPR spectra, corresponding to average of initial four EPR spectra (black) and average of final four EPR spectra (red) from the time dependent series of spectra in Fig. S4. EPR conditions and data acquisition procedures are as described in Materials and Methods.

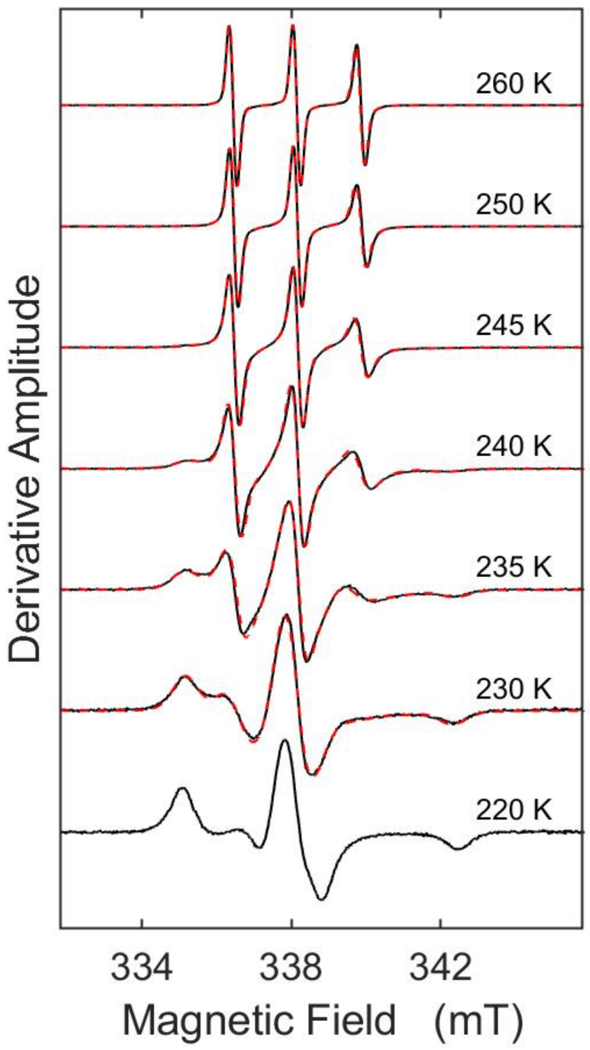

Figure S6. Temperature dependence of the TEMPOL EPR spectrum in the presence of α-synuclein (black) and overlaid two-component EPR simulations (red dashed line): α-synuclein oligomers, for increasing sequential temperature change. Rigid-limit, single-component spectra are not simulated. Spectra are normalized to the central peak-to-trough amplitude.

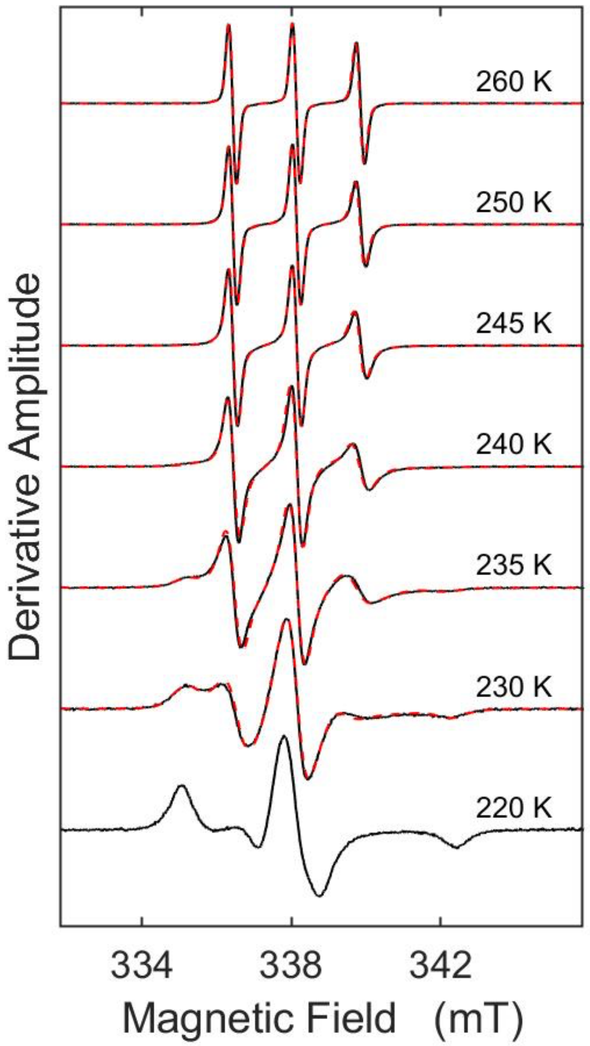

Figure S7. Temperature dependence of the TEMPOL EPR spectrum in the presence of α-synuclein (black) and overlaid two-component EPR simulations (red dashed line): α-synuclein oligomers, for decreasing sequential temperature change. Rigid-limit, single-component spectra are not simulated. Spectra are normalized to the central peak-to-trough amplitude.

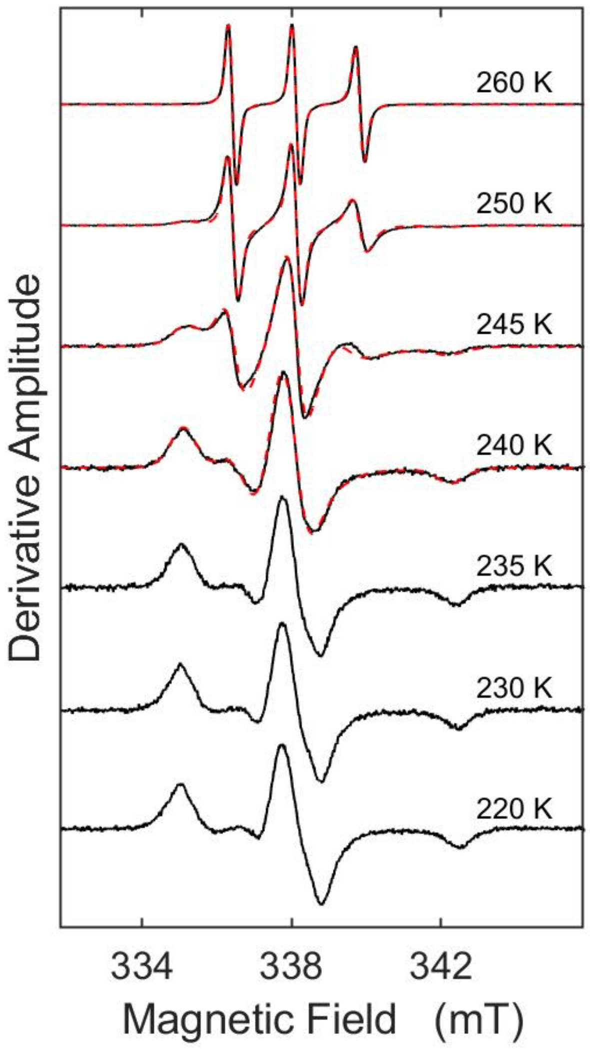

Figure S8. Temperature dependence of the TEMPOL EPR spectrum in the presence of α-synuclein (black) and overlaid two-component EPR simulations (red dashed line): α-synuclein fibrils, for increasing sequential temperature change. Rigid-limit, single-component spectra are not simulated. Spectra are normalized to the central peak-to-trough amplitude.

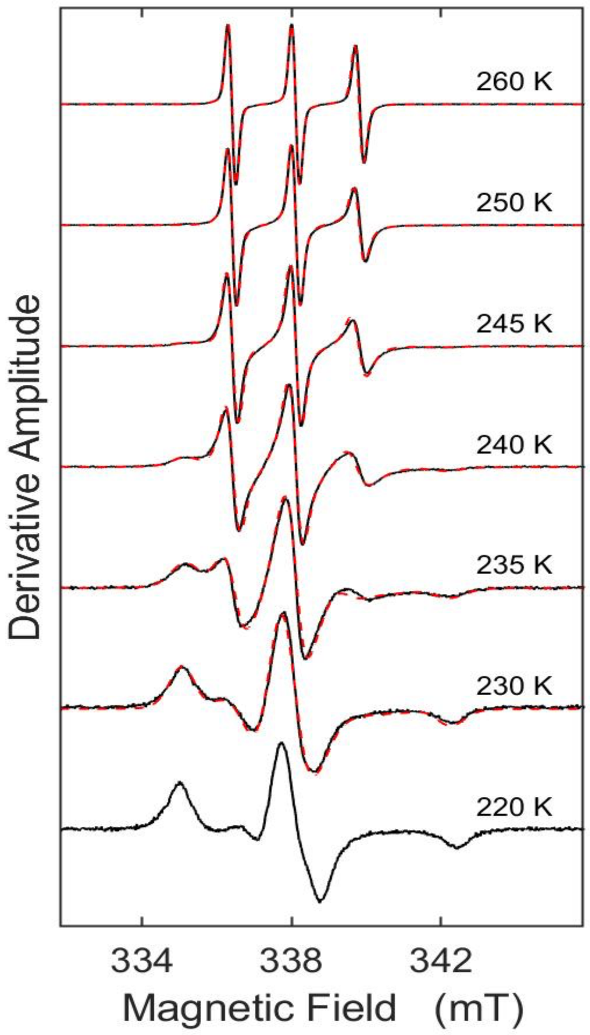

Figure S9. Temperature dependence of the TEMPOL EPR spectrum in the presence of α-synuclein (black) and overlaid two-component EPR simulations (red dashed line): α-synuclein fibrils, for decreasing sequential temperature change. Rigid-limit, single-component spectra are not simulated. Spectra are normalized to the central peak-to-trough amplitude.

SI Tables

Table S1. Mean logτ_c and *W* values at different *T* values for α-synuclein oligomers, for data collection in the direction of increasing *T*.

T **(K)**	**log $\tau_{c,s}$ (s)**	**W_s**	**log $\tau_{c,f}$ (s)**	**W_f**
225	-7.14 ± 0.08	0.91 ± 0.03	-8.41 ± 0.06	0.09 ± 0.03
230	-7.11 ± 0.12	0.74 ± 0.05	-8.44 ± 0.02	0.26 ± 0.05
235	-7.00 ± 0.01	0.57 ± 0.04	-8.58 ± 0.02	0.43 ± 0.04
240	-7.64 ± 0.09	0.47 ± 0.04	-8.87 ± 0.01	0.53 ± 0.04
245	-8.46 ± 0.19	0.38 ± 0.04	-9.15 ± 0.03	0.62 ± 0.04
250	-9.02 ± 0.03	0.28 ± 0.01	-9.42 ± 0.02	0.72 ± 0.01
255	-9.39 ± 0.09	0.23 ± 0.01	-9.62 ± 0.01	0.77 ± 0.01
260	-9.63 ± 0.02	0.21 ± 0.00	-9.82 ± 0.01	0.79 ± 0.00
265	-9.87 ± 0.03	0.22 ± 0.01	-10.04 ± 0.04	0.78 ± 0.01

Table S2. Mean logτ_c and *W* values at different *T* values for α-synuclein oligomers, for data collection in the direction of decreasing *T*.

T **(K)**	**log $\tau_{c,s}$ (s)**	**W_s**	**log $\tau_{c,f}$ (s)**	**W_f**
225	-7.20 ± 0.03	0.77 ± 0.02	-8.33 ± 0.03	0.23 ± 0.02
230	-7.38 ± 0.13	0.54 ± 0.06	-8.48 ± 0.04	0.46 ± 0.06
235	-7.67 ± 0.05	0.39 ± 0.04	-8.71 ± 0.02	0.61 ± 0.04
240	-8.24 ± 0.04	0.33 ± 0.03	-9.00 ± 0.01	0.67 ± 0.03
245	-8.78 ± 0.17	0.37 ± 0.10	-9.23 ± 0.06	0.63 ± 0.10
250	-9.00 ± 0.09	0.25 ± 0.04	-9.44 ± 0.04	0.75 ± 0.04
255	-9.33 ± 0.09	0.21 ± 0.02	-9.64 ± 0.04	0.79 ± 0.02
260	-9.60 ± 0.11	0.20 ± 0.01	-9.84 ± 0.04	0.80 ± 0.01
265	-9.80 ± 0.15	0.21 ± 0.01	-10.02 ± 0.02	0.79 ± 0.01

Table S3. Mean logτ_c and W values at different T values for α-synuclein fibrils, for data collection in the direction of increasing T.

T **(K)**	**log $\tau_{c,s}$ (s)**	**W_s**	**log $\tau_{c,f}$ (s)**	**W_f**
240	-7.18 ± 0.07	0.80 ± 0.00	-8.36 ± 0.04	0.20 ± 0.00
245	-7.49 ± 0.04	0.57 ± 0.00	-8.55 ± 0.00	0.43 ± 0.00
250	-7.84 ± 0.10	0.42 ± 0.01	-9.09 ± 0.01	0.58 ± 0.01
255	-9.19 ± 0.01	0.32 ± 0.01	-9.48 ± 0.01	0.68 ± 0.01
260	-9.55 ± 0.03	0.31 ± 0.01	-9.74 ± 0.01	0.69 ± 0.01
265	-9.82 ± 0.06	0.32 ± 0.01	-9.97 ± 0.00	0.68 ± 0.01

Table S4. Mean logτ_c and W values at different T values for α-synuclein fibrils, for data collection in the direction of decreasing T.

T **(K)**	**log $\tau_{c,s}$ (s)**	**W_s**	**log $\tau_{c,f}$ (s)**	**W_f**
230	-7.16 ± 0.03	0.78 ± 0.04	-8.41 ± 0.08	0.22 ± 0.04
235	-7.42 ± 0.02	0.64 ± 0.01	-8.53 ± 0.01	0.36 ± 0.01
240	-7.54 ± 0.03	0.45 ± 0.01	-8.81 ± 0.01	0.55 ± 0.01
245	-7.91 ± 0.04	0.38 ± 0.03	-9.11 ± 0.00	0.62 ± 0.03
250	-8.93 ± 0.01	0.32 ± 0.00	-9.34 ± 0.00	0.68 ± 0.00
255	-9.14 ± 0.01	0.25 ± 0.00	-9.56 ± 0.01	0.75 ± 0.00
260	-9.48 ± 0.03	0.23 ± 0.00	-9.79 ± 0.01	0.77 ± 0.00
265	-9.98 ± 0.15	0.34 ± 0.03	-10.38 ± 0.36	0.66 ± 0.03
